# The Effect of Balance and Coordination Exercises on Quality of Life in Older Adults: A Mini-Review

**DOI:** 10.3389/fnagi.2019.00318

**Published:** 2019-11-15

**Authors:** Ayelet Dunsky

**Affiliations:** School of Human Movement and Sport Sciences, The Academic College at Wingate, Wingate Institute, Netanya, Israel

**Keywords:** balance, coordination, exercise, older adults, quality of life

## Abstract

The ability to control balance during activities of daily living (ADL) is impaired in older adults as a result of deterioration in the sensory systems (i.e., vestibular, visual, somatosensory), the cognitive system (central nervous system), and the musculoskeletal system. Consequently, many older adults face a risk of falling during their ADL. In most cases, falls and related injuries impair the quality of life and result in physical limitations, anxiety, loss of confidence, and fear of falling. Among a variety of fall prevention interventions, adapted physical activity programs have been suggested for improving balance control during ADL. These programs challenge the sensory, cognitive, and musculoskeletal systems while addressing balance constraints such as orientation in space, changes in direction, and the speed or height of the center of mass during static and dynamic situations resembling ADL. The above-mentioned elements can be dealt with through a combination of balance and coordination exercises that challenge the postural control systems in multiple dimensions—including vertical and horizontal changes of the center of mass, standing on unstable surfaces with a reduced base of support, and changing body directions. Consequently, such exercises require environmental information-processing. The combination of dual-task, function-oriented challenges while controlling balance stimulates the sensory and neuromuscular control mechanisms. Among older adults, these programs have been found to improve static and dynamic stability, as well as a number of aspects in the quality of life. Recently, they have also been found to improve cognitive functions such as memory and spatial cognition.

## Introduction

Aging is subjected to longitudinal processes as a consequence of physiological changes, such as a higher level of stress, mitochondrial dysfunction, abnormality of inflammatory processes, decreased hormone production, and decreased metabolic rate which can lead to catabolism and degeneration of organs (Cesari et al., 2013; Sieber, 2017). These processes lead to a progressive loss of nerve extensions, bone mass, skeletal muscle mass, and strength (Chang and Lin, 2015; Sieber, 2017; Nascimento et al., 2019). Consequently, frailty and sarcopenia may be present in approximately 10% of people over the age of 65 and 25–50% of those aged over 85 (World Health Organization, 2007). These are phenomena known to impair the ability to perform activities of daily living (ADL), reduce the quality of life, and increase the risk of falling (Chang and Lin, 2015; Landi et al., 2015; Sieber, 2017; Nascimento et al., 2019).

The increased risk of falling, shown to be a consequence of the above-mentioned processes, is mainly due to the difficulties of older adults in maintaining postural control while performing ADL (Rubenstein, 2006; Kojima, 2015). Postural control is based on the ability to synchronize several systems in an ongoing cycle: the sensory systems (i.e., vestibular, visual, somatosensory), the cognitive system (central nervous system), and the musculoskeletal system. During normal aging, physiological changes occur in one’s visual, vestibular, and somatosensory inputs, as well as in the central processing and muscular effectors (Horak, 2006; Rubenstein, 2006). In the visual system, a reduced ability to detect low contrast hazards, judge distances, and perceive spatial relationships appears to be the significant impairment associated with falls among older adults (Lord, 2006). Changes in the vestibular system are expressed in a reduced number of hair cells in the semicircular canals, in the maculae of the saccule and the utricle, as well as in the primary and secondary vestibular neurons (Ishiyama, 2009). The reduced capacity to detect position and direction of movement, together with reduced lower-limb strength and sensation, are considered to be significant predictors of fall risk among older adults (Lord et al., 1996). Moreover, inter-joint coordination and the appropriate timing of muscle action during ADL, such as in walking, is also affected; thus, the ability of older people to use the fall avoidance strategies practiced by young people is reduced (Rubenstein, 2006).

Several therapeutic approaches have been shown to be effective for fall prevention among older adults, and there is a large body of literature documenting the favorable effects of physical activity training programs. Evidence suggests that programs based on aerobic and resistance exercise can be used to restore or maintain functional independence in older adults, and may also potentially prevent, delay, or reverse frailty (de Vries et al., 2012; de Labra et al., 2015). Aerobic training enhances cardiovascular function, as it was shown to preserve motor units and mitochondrial function. Additionally, it was found to prevent muscle atrophy and to improve the health-related quality of life (Navas-Enamorado et al., 2017). Resistance training was shown to increase protein synthesis and muscle mass, as well as to improve neural recruitment and muscle strength, explained by neural and morphological adaptations (Guizelini et al., 2018). The combination of these training modes has yielded beneficial effects in body composition and physical function, as well as in cognitive and emotional function, among frail patients (de Labra et al., 2015; Tarazona-Santabalbina et al., 2016). It is important to note that in most cases aerobic and strength exercises require the participants to be highly mobile. Older adults with low mobility might have difficulty in benefiting from these exercises because of their limited locomotive ability. Therefore, exercise with reduced locomotion requirements, such as balance or coordination exercise, may provide similar benefits to older adults with a variety of mobility abilities (Kwok et al., 2011).

The possibility of improving the quality of life of older people through simple exercises can be a solution for people with a wide range of functional abilities. Thus, the current mini-review has two purposes: (a) to present studies examining the effect of performing balance and coordination exercises on various aspects of the quality of life of older people; and (b) to present suggestions for practical applications.

## Methods

**Figure 1:**
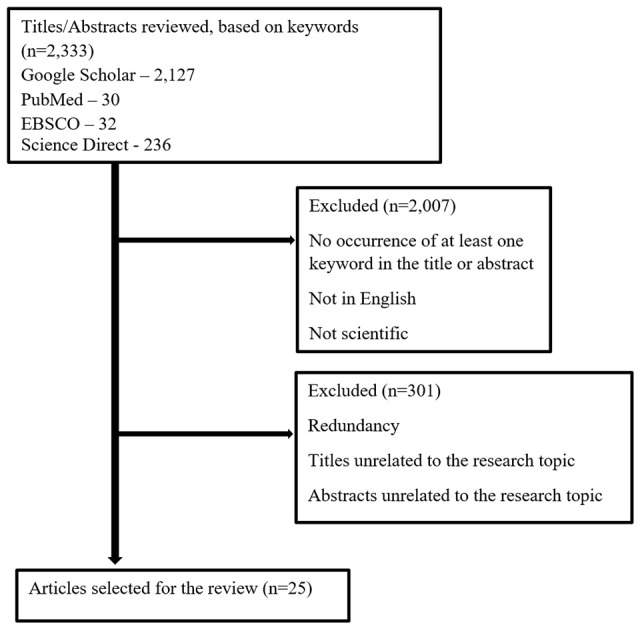
Flowchart of study selection.

The author performed a literature review of available studies on the research topic dealing with different effects of balance and/or coordination exercise programs on various aspects of older adults’ lives. Research studies were selected on the basis of research topics that included the following keywords: balance exercise, coordination exercise, postural control, risk of falling, frailty, and quality of life, found in the world’s acknowledged databases: PubMed, EBSCO (SPORTDiscus, Academic Search Complete, EDS), Google Scholar, and Science Direct. The search was not limited by any period of time. From the database/journal searches, 2,333 titles/abstracts were retrieved. The majority of the studies were detected in the Google Scholar database. The titles and abstracts of some identified articles were then checked for relevance. Subsequently, the search was performed again, focusing on the occurrence of at least one keyword in the title or abstract of articles written in English and representing scientific study, thereby significantly narrowing down the selection. Altogether, 326 studies were found. After removing duplicates and titles/abstracts unrelated to the research topic, 25 studies were found to be relevant to the research topic (see Figure 1 for the flowchart of study selection). The information found in the selected studies on balance and coordination exercise and its effect on postural control, cognitive function, and the quality of life of older adults was evaluated, and it is summarized in Table 1 and described and discussed in the following sections.

**Table 1:** Description of studies that included balance and/or coordination and changes in postural control, cognitive performance, or quality of life among adults.

Study	Participants	Intervention/ Assessments	Outcome measures	Major findings
**BALANCE AND POSTURAL CONTROL**
Bisson et al. (2007)	Older adults *n* = 24, Age: 74 Study groups: 1. Virtual reality (VR; *n* = 12) 2. Biofeedback (*n* = 12)	Training program: 20 sessions over 10 weeks of dynamic balance exercises using VR (“juggling” a virtual ball while standing), or dynamic balance exercises with visual biofeedback (moving a curser that represents Center of Pressure (COP) while standing on force plate)	Static balance—sways of COP in different postures; Simple reaction time task—while maintaining balance; Functional balance and mobility—the Community Balance and Mobility Scale (CB&M)	After a 10-week intervention, both groups significantly improved their functional balance and mobility, as well as their reaction time during standing.
de Vries et al. (2018)	Older adults *n* = 30, Age: 70 Young adults *n* = 30, Age: 22 Study conditions: 1. FLOS task 2. VR—Kinski 3. VR—Wiiski	One session of VR game: steer an avatar skiing down a slalom track	Time to complete the game; Maximum displacement of Center of Mass (COM); Peak COM speed	COM displacement during the Kinski was significantly larger in all directions compared to the Wiiski. Peak COM speed was significantly higher in the Kinski.
Dunsky et al. (2017b)	Older adults *n* = 112, Age: 75 Study groups: 1. women *n* = 862. men *n* = 36	Associations between static balance and dynamic balance	TUG; FR; COP length of sway; COP sway intensities	In general: low correlations were found between static and dynamic balance measures, for both women and men
Nagy et al. (2007)	Older adults *n* = 19, Age: 79 Young adults *n* = 11, Age: 22 Study groups: 1. training (*n* = 9) 2. control (*n* = 10) 3. young adults (*n* = 11)	Training program: 16 sessions over 8-weeks, static and dynamic balance, strength, flexibility and aerobic Control: routine	COP path and frequencies (AP, ML) during standing with eyes open and eyes closed. TUG test	Training group: Improved postural control in the ML direction. Improved performance on TUG test
Rendon et al. (2012)	Older adults *n* = 40, Age: 60–95 Study groups: 1. virtual reality (VR) 2. control	Training program: 18 session over 6 weeks of VR balance games Control: routine	Dynamic balance– 8 feet up and go test; Balance confidence—Activities–specific Balance Confidence Scale (ABC); Depression—Geriatric Depression Scale	After 6 weeks of intervention, the VR group showed significant improvement in the dynamic balance test and in the ABC score compared with the control group.
**BALANCE AND COGNITIVE PERFORMANCE**
Mouthon and Taube (2019)	Young adults *n* = 26, Age: 24 Study groups: 1. training (*n* = 13) 2. control (*n* = 13)	Training program: six sessions, over 2-weeks, balancing on a movable platform. Control: routine physical activity	Time kept in horizontal position on a movable platform; EMG of tibialis anterior and soleus muscles; Short-interval intracortical inhibition (SICI)	The balance training led to an increased intracortical inhibition during balance tasks, as well as improved balance performance, with reduced EMG activities during unstable conditions.
Netz et al. (2018)	Older adults *n* = 112, Age: 77 Study groups: 1. young women (*n* = 41, age: 69) 2. old women (*n* = 38, age: 79) 3. men (*n* = 33, age: 77)	Associations between static and dynamic balance and attention inhibition	TUG; FR; COP length of sway; COP sway intensities; Computerized go/no-go test	Attention inhibition was significantly correlated to static balance for young women, to dynamic balance for men, and not correlated to balance for older women.
Rogge et al. (2017)	Adults *n* = 40, Age: 18–65 Study groups: 1. balance (*n* = 19) 2. relaxation (*n* = 21)	Training program: 24 sessions over 12-weeks, Balance circuit training in eight stations. Or Relaxation training using progressive muscle relaxation and autogenic training.	Dynamic balance—platform time at horizontal position; BESS; COP sway velocity; Cardiorespiratory fitness; Memory—auditory verbal paired associated learning task; Spatial cognition—Orienting and Perspective Taking test; Figure orientation; Mirror Images; Stroop test	The balance training improved participants’ dynamic balance. Significantly higher memory scores for the balance training group. No changes were found in the BESS or the cardiorespiratory fitness.
Rogge et al. (2018)	Adults *n* = 37, Age: 19–65 Study groups: 1. balance (*n* = 19) 2. relaxation (*n* = 18)	Training program: 24 sessions over 12-weeks, Balance circuit training in eight stations. Or: Relaxion training using progressive muscle relaxation and autogenic training.	Dynamic balance—platform time at horizontal position; MRI Cortical thickness; Subcortical gray matter volume	The balance training group had significantly improved balance performance, and showed significantly higher cortical thickness increases following the intervention.
**BALANCE TRAINING AND QUALITY OF LIFE**
Gouveia et al. (2018)	Older adults *n* = 52, Age: 65–85 Study groups: 1. intervention (*n* = 26) 2. control (*n* = 26)	Training program: 24 sessions over 12-weeks, gait, balance, functional training, strengthening, flexibility and 3D training	SF-36 questionnaire	Significant improved quality of life for the intervention group.
Halvarsson et al. (2011)	Older adults *n* = 59, Age: 67–93 Study groups: 1. intervention (*n* = 38) 2. control (*n* = 21)	Training program: 36 sessions over 12-weeks, progressive and specific balance exercises for ADL Control: regular life during the study period.	Falls Efficacy Scale International (FES-I); Reaction time of step execution; Gait—spatio-temporal variables	Intervention group showed significant improvements in FES-I, in reaction time parameters and several gait parameters. In general—it led to decreased fear of falling.
Halvarsson et al. (2013)	Older adults *n* = 59, Age: 67–93 Study groups: 1. intervention (*n* = 38) 2. control (*n* = 21)	Training program: 36 sessions over 12-weeks, progressive and specific balance exercises for ADL Control: regular life during the study period.	Gait speed; Step execution; Fear of falling; likelihood of depression	Gait speed, step execution and fear of falling were still improved in the intervention group at 9-months follow-up. At 15-months follow-up, only fear of falling was significantly improved. Other parameters were significantly better compared to the control group.
Halvarsson et al. (2015a)	Older adults Age: 66–89	Training program: 36 sessions over 12-weeks, balance demanding exercises at three levels of progression	FES-I; Fear of falling; Gait speed with and without cognitive task; Balance performance; physical function	All intervention groups had significantly better scores in FES-I, walking speed with dual-task, balance performance and lower extremities’ function, and reduced fear of falling compared to the control group.
Halvarsson et al. (2015b)	Older adults with osteoporosis *n* = 96, Age: 66–87 Study groups: 1. training (*n* = 34) 2. training + physical activity (*n* = 31) 3. control (*n* = 31)	Training program: 36 sessions over 12-weeks, progressive balance with dual and multi-task, and physical activity	FES-I; Fear of falling; Gait speed with and without cognitive task; Balance performance; physical function	Both intervention groups had significantly better scores in FES-I, walking speed with dual-task, balance performance and lower extremities’ function, compared to the control group.
Taguchi et al. (2010)	Older adults *n* = 65, Age: 74–96 Study groups: 1. intervention (*n* = 31) 2. control (*n* = 34)	Training program: one session per week for 12-months of various exercise related to flexibility, strength, aerobic and balance	Lower limb strength; Sit-and-reach test; Grip strength; 6-min walking; Falls Efficacy Scale; MMSE; IADL	After 12 months of intervention, the intervention group had significant improvement in lower-limb strength, sit-and-reach test, as well as in the Falls Efficacy Scale, representing improvement in quality of life.
**COORDINATION TRAINING AND BALANCE PERFORMANCE**
Lelard et al. (2010)	Older adults *n* = 28, Age: 70–85 Study groups: 1. Tai-Chi (*n* = 14) 2. balance training (*n* = 14)	Training program: 24 sessions over 12-weeks, of 10 Tai-chi forms adapted for older adults, Or balance exercises that involved shifting the body part (or COM) in different positions.	Static postural control: COP sways with eyes open and closed; Walking speed over 10-m course.	After a 12-week intervention no significant differences were found in walking speed or postural control for both groups.
Wong et al. (2001)	Older adults *n* = 39, Age: 66–76 Study groups: 1. Tai-Chi (*n* = 25) 2. control (*n* = 14)	The Thai-Chi group practiced Thai-Chi for 2–35 years	Static postural stability; Dynamic balance test	The Thai-Chi group had significantly better results in complication static conditions (eyes closed, sway surface), as well as in one of the dynamic balance tests, compared to the control.
**COORDINATION AND COGNITIVE FUNCTION**
Gao et al. (1996)	Adults *n* = 6	Performance of four different tasks: passive and active cutaneous discrimination tasks, active grasp objects task with two hands and coordinated finger movements	Dentate nuclei activation using MRI	The highest cerebellar activity was find during the coordinative activity. Dentate activation was greatly enhanced when sensory discrimination was paired with finger movements.
Kwok et al. (2011)	Older adults *n* = 40, Age: 66–90 Study groups: 1. coordination group (*n* = 20) 2. towel exercises group (*n* = 20)	Training program: 8 sessions over 8-week of simple coordination exercises, Or stretching exercises using a towel, mainly training upper limbs.	Two cognitive function assessments: Chinese Mini-Mental State Examination; Chinese Dementia Rating Scale (CDRS); TUG	After an 8-week intervention, the CDRS scores of the coordination group improved significantly.
Niemann et al. (2014)	Older adults *n* = 49, Age: 62–79 Study groups: 1. cardiovascular training (*n* = 17) 2. coordination training (*n* = 19) 3. control (*n* = 13)	Training program: three sessions per week for 12 months of Nordic Walking program (for the cardiovascular group), Or: eye-hand and leg-arm coordination, spatial orientation and reaction to moving objects exercises (for the coordination group), Or: stretching and relaxation training (for the control group).	After 12-months oc fitness significantly improved in the cardiovascular group; action speed performance and hippocampal volume significantly improved in both cardiovascular and the coordination groups.	
Voelcker-Rehage et al. (2011)	Older adults *n* = 44, Age: 62–79 Study groups: 1. cardiovascular training (*n* = 17) 2. coordination training (*n* = 19) 3. control (*n* = 13)	Training program: three sessions per week for 12 months of Nordic Walking program (for the cardiovascular group), Or: eye-hand and leg-arm coordination, spatial orientation and reaction to moving objects exercises (for the coordination group), Or: stretching and relaxation training (for the control group).	Functional MRI—changes in brain activation patterns; Executive function; Perceptual speed	After 12-months of intervention both cardiovascular and coordination groups improved in executive function and perceptual speed. In addition, brain activity patterns changed, indicating more efficient information processing.
**THE COMBINATION OF BALANCE AND COORDINATION EXERCISE**
Dizdar et al. (2018)	Older adults, postmenopaus-al females with osteoporosis *n* = 68, Age: 50–75 Study groups: 1. balance and coordination (*n* = 25) 2. strengthening (*n* = 25) 3. aerobic (*n* = 25)	Training program: 36 sessions over 12 weeks, of balance and coordination exercises, Or strengthening exercises on abdominals and back muscles and upper and lower extremities, Or aerobic training by walking on treadmill.	Static balance (COP sways); Dynamic balance: TUG and Berg Balance Scale; Pain assessment; Life quality assessment	After 12 weeks of intervention the balance and coordination group significantly improved in static and dynamic balance performances. Both balance and coordination and strengthening groups showed improvement in general health, however, the strengthening group had significant improvement in terms of mental function compared to the other groups.
Dunsky et al. (2017a)	Older adults *n* = 36, Age: 72 Study groups: 1. step aerobics (SA; *n* = 14) 2. stability ball (SB; *n* = 13) 3. control (*n* = 9)	Training program: 16 sessions over 8 weeks, of strength, balance and coordination exercises using aerobic steps, or stability ball. Control: ceramic sculpture class	Balance assessments: TUG, One-Leg Stand test, FR, The Tinetti Performance-Oriented Mobility Assessment (POMA); Quality of life assessment—The Short Form-36 Health Survey questionnaire (SF-36)	After an 8-week intervention the SA group significantly improved their TUG and POMA performances; General health perception improved significantly among both SA and SB groups compared to the control.
Segev et al. (2019)	Older adults with cardio-vascular diseases *n* = 26, Age: 74 Study groups: 1. intervention (*n* = 13) 2. control (*n* = 13)	Training program: 24 sessions over 12-weeks, balance and coordination exercises within 20 min of warm-up. As part of 80 min physical activity for cardiac rehabilitation. Control: Traditional warm-up	TUG test FR test BESS FTSST	Significant improvement in TUG, BESS and FTSST only in the intervention group
Taylor-Piliae et al. (2006)	Older adults with cardio-vascular diseases *n* = 39, Age: 66	Training program: 36 session over 12 weeks of Tai Chi 24 postures	Mood state (POMS); The Perceived Stress Scale test; Tai-Chi exercise self-efficacy	Significant improvements in all measures of psychosocial status were found following the intervention, as well as increased Tai-Chi self-efficacy.

*BESS, Balance Error Scoring System; COM, Center of Mass; COP, Center of Pressure; FLOS, Functional Limits of Stability; FR, Functional Reach; FTSST, Five Time Sit to Stand Test; TUG, Time Up and Go; VR, Virtual Reality; POMA, The Tinetti Performance-Oriented Mobility Assessment*.

## Balance and Postural Control

During ADL people are susceptible to changes in both dynamic as well as static balance. The ability to control those changes represents a complex challenge for the neuromuscular control system, which must cope with rapid environmental changes and is based upon the ability to proact or react to these changes for successful locomotion and fall prevention. This challenge can be met if there is proper function of the visual, vestibular, proprioceptive, and tactile senses for correct sensory input, and if they work together with the neuromuscular system to control body alignment with the correct subsequent motor output (Hayes, 1982; Horak, 2006; Dunsky et al., 2017b). The proactive balance strategy activates postural adjustments prior to the occurrence of destabilizing forces upon the body. The reactive balance strategy activates postural adjustments after an external disturbance is encountered, thus assuring balance recovery (Hayes, 1982; Wong et al., 2001). As people age, their ability to use these strategies—and in particular the reactive balance strategy—is impaired, as a consequence of the physiological and cognitive changes mentioned above. Thus, specific training programs that are based on postural control exercises are suggested for older adults (Arampatzis et al., 2011). If the purpose of training is to evoke improvements in reactive balance, then the training program may be associated with all postural control systems, including the musculoskeletal system, the cognitive system, as well as the somatosensory feedback system, while challenging the body in different environmental situations and unexpected perturbations (Tinetti et al., 1986; Robbins et al., 1989; Arampatzis et al., 2011). During balance training, participants perform exercises that include static vs. dynamic stability postures, reducing the base of support (bipedal vs. tandem vs. one-leg stance), changes in the height of the center of gravity, changes in the standing surface (such as floor, wobble boards, wobble cushions, foam, or perturbation platforms), and reducing the source of visual information, while attempting to simulate perturbations leading to falls during daily activities (e.g., eyes open vs. closed; Zech et al., 2010; Sibley and Salbach, 2015; Rogge et al., 2018). Nagy et al. (2007) found that an 8-week program that included static and dynamic balance exercises combined with strength, flexibility, and aerobic exercises, improved the postural control of older adult participants, especially in the more challenging direction (i.e., mediolateral), with and without visual control. The authors suggested that improvement was a consequence of the establishment of a new postural control strategy that was developed by the participants as a result of the specific balance intervention. de Vries et al. (2018) suggested using virtual reality training for postural control improvement, as it may challenge all aspects of the balance control system. Virtual reality can incorporate anticipatory postural adjustments, postural responses, muscle loading, cognitive challenges, transitioning into different poses, and taking steps. The authors examined two types of virtual reality training of skiing and found that the Kinski game elicited a larger center of mass displacements than the Wiiski game, and thus suggested the Kinski as a better method of balance training for older adults. In another study, an interventional program of 20 sessions of virtual reality was shown to improve functional balance and mobility among older adults, with results similar to biofeedback training (Bisson et al., 2007). Additionally, a training program of 18 sessions of virtual reality was shown to improve dynamic balance and balance confidence, in comparison to the control group (routine activities) older adults (Rendon et al., 2012).

The use of balance training for postural control is only one aspect of quality of life improvement. It has also been suggested as beneficial for cognitive function beneficial for cognitive function improvement.

## Balance and Cognitive Performance

Physical activity, and in particular aerobic exercise, has been discussed as a promising means for increasing neurogenesis and plasticity of the brain, in order to improve cognitive functions as well as to protect against the age-related decline in the ability of the brain to adapt to environmental demands (Stimpson et al., 2018). As balance training provides a stimulus to the vestibular, neuromuscular, and proprioceptive systems, which then send signals to specific areas in the brain that make connections between vestibular nuclei and the cerebellum, hippocampus, as well as prefrontal and parietal cortices, it may affect cognitive functions such as spatial functions, navigation, and memory (Taube et al., 2007; Smith and Zheng, 2013). It has been speculated that increased stimulation of the vestibular system during balance exercise may be a mediator between physical exercise and cognitive functioning (Smith et al., 2010). In a cross-sectional study, Netz et al. (2018) found that attention inhibition was correlated to static balance among older women, while in older men it was associated with dynamic balance. Based on their results, the authors recommended that men include static balance exercises in their exercise routine and that women include dynamic exercise.

Rogge et al. (2017) found that 12 weeks of balance training improved memory and spatial cognition among healthy adults. In addition, they found increased cortical thickness in specific regions of the brain (i.e., the superior temporal cortex, visual association cortices, the posterior cingulate cortex, the superior frontal sulcus, and in the precentral gyri), among the same group. These changes were found to be correlated with improved balance performances (Rogge et al., 2018). The authors suggested that the brain’s regions that show changes play a role in spatial orienting and memory, stimulating visual-vestibular pathways during self-motion, and thus they may mediate the beneficial effects of balance exercise on cognition. Mouthon and Taube (2019) found that 2 weeks of balance training on an unstable platform improved postural control that was correlated with improvements in intracortical inhibition. The authors suggested that these changes may demonstrate the occurrence of cortical plasticity and adaptation of inhibitory behavior for the acquisition of a balance task following the balance training intervention.

## Balance Training and Quality of Life

Gouveia et al. (2018) found that 12 weeks of a rehabilitation program that included gait, balance, functional training, strengthening, and flexibility training in community-dwelling older adults can lead to significant improvements in multiple aspects of quality of life (assessed by the Short Form Health Survey—SF-36). The authors suggested that this improvement was based on the enhanced balance performance, as well as on the educational aspect of the program that was used in their study, since quality of life is strongly associated with both physical and mental age-related factors. Taguchi et al. (2010) found that a 12-months program of various exercises related to flexibility, strength, aerobic and balance significantly improved lower-limb strength as well as fall efficacy scale, but not other measures of quality of life. Halvarsson et al. (2011) found that individually adjusted, progressive, and specific balance group training for 3 months positively affected the fear of falling of community-dwelling elderly people. The authors mentioned that exercise in groups provides a social belonging which may contribute to an increased attendance rate, and thus can also influence the domain participation and improve the quality of life through increased activity in daily life (Lelard and Ahmaidi, 2015). In several continuous studies, Halvarsson et al. (2013, 2015a,b) found that this same balance progressive program improved gait speed and lower-limb strength reduced the likelihood of depression and improved balance confidence among older adults.

## Coordination Training and Cognitive Function

Coordination exercise with low velocity, low impact, and a high-interest level, which also provides a good training effect, is preferred for most older persons (Wong et al., 2001). These exercises were associated with high activation in visual-spatial networks in the brain of older adults (Voelcker-Rehage et al., 2011; Niemann et al., 2014). Coordinative exercise is known to involve an activation of the cerebellum (Gao et al., 1996), which is responsible for motor control and motor learning (Manto et al., 2012), and was also found to influence a variety of higher cognitive functions, including divided attention and working memory (Gottwald et al., 2003), and verbal learning and memory (Tomlinson et al., 2014). In addition, bimanual coordination movements have been shown to lead to activation in the pre-frontal cortex, specifically the medial frontal region, which is also involved in attention to demanding cognitive tasks, spatial memory, self-initiated movement, and conflict resolution (Spinella et al., 2004).

An 8-week coordination training program based on 11 movements, including coordination of fingers, hands, eyes, and legs while the participant is sitting (which is a simplified version of Tai Chi), significantly improved cognitive function (as assessed by the Chinese Dementia Rating Scale) of older adults (Kwok et al., 2011). Voelcker-Rehage et al. (2011) and Niemann et al. (2014) found that 12 months of coordination training was associated with increased activation in the visual-spatial network, and led to changes in the total hippocampal volume of older adults. The authors suggested that this result was a consequence of the fact that to a high degree coordinative exercises rely on and practice spatial orientation, and the hippocampus is known to be involved in spatial memory processes.

## The Combination of Balance and Coordination Exercise

As coordination exercise was suggested to improve cognitive functions, and balance exercise was shown to improve balance, cognitive functions, as well as quality of life, it was suggested that the combination of balance and coordination exercise may result in greater improvement in the quality of life among the elderly. One example of such a combination is suggested by Tai Chi exercises, which are based on a series of movements linked together in a continuous sequence (i.e., coordination) while the body is constantly shifting from foot to foot, with the knees and hips held in flexion (i.e., balance). During each movement, different parts of the body take turns playing the role of stabilizer and mover, allowing smooth movements to be executed without compromising balance and stability. Older adults who performed this form of exercise were found to have significantly better postural control and stability in conditions with simultaneous disturbance of vision and proprioception, compared to active nonpractitioners (Wong et al., 2001). This form of exercise was also examined in a 12-week Tai Chi exercise program and has been found effective in reducing perceived stress and improving mood state, as well as increasing perceived social support (Taylor-Piliae et al., 2006). On the contrary, 24 sessions of Tai Chi exercises during a 12-week intervention did not improve static postural control or walking speed among older adults (Lelard et al., 2010).

Another example of the combination of balance and coordination exercises, together with resistance and aerobic training, was suggested by Dizdar et al. (2018) for fall prevention among women with osteoporosis. The part of balance and coordination in the exercises included: single leg stances with eyes open and closed, tandem stance, toe walking, heel walking, tandem gait, reciprocal lower extremity movement, half-squatting, bridging, modified Romberg exercise on hard and soft ground with eyes closed, edge walking, walking on a balance board, reciprocal leg movements, slowly sitting down and standing up from a chair, and going up and down the stairs. The authors found that 12 weeks of this combined program improved the participants’ quality of life (as assessed by the Quality of Life Questionnaire of the European Foundation for Osteoporosis). The authors suggested that this improvement may be the result of performing exercises as a group, which enhanced the social aspect of the participant (Dizdar et al., 2018). This combination was also studied by Dunsky et al. (2017a), who found that 8 weeks of balance training combined with coordination by the means of dual-task exercises had a significant positive effect on the quality of life (as assessed by the Short Form Health Survey—SF-36) of community-dwelling older adults. In this study, two balance programs were conducted: step aerobics—while using the step as an obstacle (moving near and around it, stepping on it, adding music while walking, and adding dual-task exercises and resistance exercises while walking around and on the step); and the stability ball—while using the ball as an unstable surface (sitting or lying on the ball while performing resistance exercises, and adding music and adding dual-task exercises while having postural control of the body on the ball). The authors suggested that the improved quality of life was probably the consequence of high compliance to the intervention in both groups, which led their participants to feel more comfortable in performing ADL and about other aspects of life, thus leading to a higher general health perception at the end of the study. Recently, Segev et al. (2019) studied the effect of balance and coordination exercises that are incorporated within a traditional cardiac-rehabilitation program for older adults with cardiovascular diseases. Following a 12-week program in which they trained twice a week, the participants of the intervention group improved their static and dynamic balance, as well as their functional strength. These skills are considered major components in ADL, and thus their improvement may imply a better quality of life.

## Discussion and Conclusion

The current mini-review points to the advantages of a combination of balance and coordination exercise on behavioral and neurophysiological outcomes among older adults. Based on the above literature, it is suggested that older adults be exposed to a program that includes such a combination for 2–3 sessions each week, for periods of at least 8 weeks, as a tool for quality of life improvement. Instructors or clinicians who wish to include such combination exercises in a program for older adults should introduce them gradually, allowing for the proper adjustment of the trainees while ensuring their safety. The program should incorporate exercises that include static vs. dynamic stability postures, changes in the base of support, variations in the height of the center of gravity, and different standing surfaces. Additionally, it should progressively reach higher levels of challenges in the form of more complex exercises involving both motor and cognitive tasks (dual- and multi-task activities). When planning each training session, it is important to keep in mind the participants’ adaptation process to the exercises and to determine the session’s optimum duration, allowing for gradual and safe exposure to new equipment or a new exercise.

## Author Contributions

AD performed the drafting, analyses, and final version of the entire manuscript.

## Conflict of Interest

The author declares that the research was conducted in the absence of any commercial or financial relationships that could be construed as a potential conflict of interest.
